# Chaos to clarity: interpreting time series complexity metrics with an application to depression

**DOI:** 10.1007/s44192-025-00231-4

**Published:** 2025-07-01

**Authors:** Sandip V. George

**Affiliations:** Department of Physics, Institute for Complex Systems and Mathematical Biology, University of Aberdeen, Aberdeen, UK

**Keywords:** Complexity, Entropy, Time series, Depression, Psychopathology

## Abstract

There is an increasing understanding in recent years that mental health and psychiatric illnesses can be interpreted as complex dynamical systems. This understanding is largely derived from the complexity of dynamics that is observed in time series that are closely related to mental health. This complexity is quantified using a range of metrics from information theory and nonlinear time series analysis. Interpreting these metrics correctly and discerning how they vary as the nature of the dynamics changes is important to correctly identify the effect of mental illness. In this perspective article I attempt to do this, by first describing complexity of time series and the metrics that are used to quantify this complexity. I then analyze the behavior of these metrics as dynamics transition between periodicity, chaos and noise. Finally, I explore these changes in the context of depression by studying existing literature, and interpret what this implies for the nature of its underlying dynamics. There are divergent trends across studies and across domains such as actigraphy, EEG, and ECG. These findings emphasize the need for a nuanced interpretation of complexity metrics and their role in advancing our understanding of the nonlinear dynamics underlying mental health conditions like depression.

**Supplementary Information:**

The online version contains supplementary material available at 10.1007/s44192-025-00231-4.

## Introduction

Individuals suffering from mental illnesses show unpredictable and complex trajectories in symptoms. This has contributed to the view that these illnesses can be understood as complex dynamical systems [1–4]. Dynamical systems are characterized by their ability to show rich and complex dynamical behavior, including sensitivity to initial conditions and sudden transitions. These systems are governed by differential equations, that can be deterministic, stochastic or a combination of the two. When the dynamics is deterministic, they can often be approximated by a few variables and represented in a low dimensional space.

The dynamics of real-world systems can often be approximated using such dynamical models, and are understood through time series analysis, where the time series of one or more of the variables of the system is studied. These time series exhibit a range of temporal scales, leading to complex dynamics. The field of nonlinear time series analysis consists of a range of tools to study the properties of time series from the point of view that the time series is derived from a dynamical system. Common techniques that are used in nonlinear time series analysis include recurrence quantification analysis (RQA), fractal dimensions, Lyapunov exponents etc. Since the time series can show a range of temporal scales, spectral analysis and information theory metrics are also often employed to study them. However, many of these metrics quantify different aspects of complexity and vary in different ways when the underlying dynamics change.

Various time series that are known to be related to psychopathology are often studied to better understand underlying patterns of psychiatric illnesses. These include time series of physical activity, the electrocardiogram (ECG), the electroencephalogram (EEG) and ecological momentary assessment (EMA). Many of these time series have been shown to have nonlinear dynamics, showing fractal structures and rich information content [5–9].

A specific pathology that has been widely studied in the context of complexity is depression. Depression is a common mental disorder that is a leading cause of disability, worldwide. It is thought to affect around 5% of the adult population [10, 11]. In this paper, we use the term depression broadly to refer to unipolar depressive disorders, including both single and recurrent episodes (ICD-10 codes F32.1–F32.9, F33.1–F33.9). Though categorized under one umbrella, it is well known that depression manifests differently between individuals, extensively varying in severity and course of development. Individuals often experience unpredictable fluctuations in their symptom severity making the treatment of depression quite challenging [12]. This makes understanding the dynamics of depression an important step towards effective treatment strategies.

In the following sections we discuss what is meant by the complexity of a time series. We then describe the metrics that are used to quantify complexity. We then examine how these metrics vary as the dynamics become more noisy and more periodic. We finally describe how these metrics have been used in the context of different measurements of depression, and attempt to interpret what this implies for the difference in the dynamics of that measurement.

## What is complexity

The primary question that comes into the picture when describing changes in complexity is how we define it. The term is rather loosely defined, with differing meanings in different fields [13]. We initially present a brief overview of the differing definitions of complexity and how they relate to each other.

**Fig. 1:**
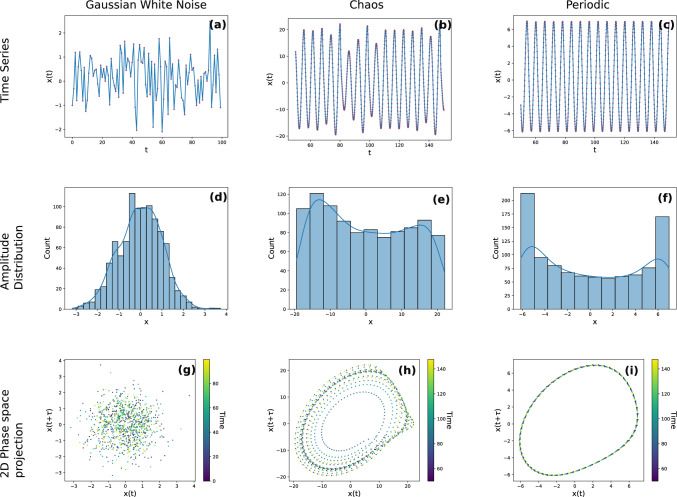
**a-c** Time series, **d**-**f**amplitude distributions and **g**-**i **2-d phase space projections from white noise **a,d,g**, chaotic **b,e,h** and periodic systems **c,f,i**

In the context of time series, complexity broadly relates to the number and relationships between the temporal scales present in it. At the extremes are time series that are composed of a single period, and time series composed of an infinite number of unrelated periods. An example of the former is a sine wave, while the latter is seen in random white noise, where every point is unrelated to the previous one. We can fully determine how a periodic time series will behave in future, when we have observed atleast one full oscillation of the system. On the other hand, every point in a random noise process is completely unrelated to the previous points. Between these extremes lie time series that arise from deterministic chaos, where the dynamics is composed of multiple frequencies that are related to each other [14]. Real time series are a combination of these, with deterministic and stochastic components evolving under certain evolution rules. Distinguishing between these components is a highly non-trivial problem, and is at the crux of determining the complexity of a time series. Figure 1 (a)-(f) shows the time series and corresponding amplitude distributions of noisy, chaotic and periodic time series.

Different metrics quantify different aspects of time series complexity, and can become high due to multiple reasons. For instance purely periodic processes with added noise can be easily mistaken for deterministic chaos [15]. Below we list a range of different metrics which are used to calculate time series complexity, broadly grouped into information theory based metrics and dynamical systems based metrics [16].

### Metrics based on information theory

One of the most popular definitions of complexity is information complexity which is based on the information content in a time series. The simplest information theory metrics, such as Shannon entropy, are not time-dependent, and characterize only the amplitude distribution of the time series.

The Shannon entropy is the starting point for most information theory metrics, and is related to the probability of occurrence of a particular value in the time series. If all values occur with equal probability in the time series, the Shannon entropy will be high. This would be the case for time series with amplitude distributions that are relatively flatter [17]. On the other hand, Shannon entropy is low if a few values dominate.

The approximate entropy (ApEn) and Sample entropy (SampEn) quantify the unpredictability of fluctuations in a time series by analyzing repeated patterns in it. This is done by first constructing time delayed or template vectors in a time series, i.e. $\vec {v_i}=(x_i,x_{i+1},...,x_{i+m-1})$. To quantify repetitions in patterns in this space, we need to examine when two template vectors are within a neighborhood of each other. Hence, the number of constructed vectors that are within a certain threshold distance,$\epsilon$, from each other are counted, $C_i^m(\epsilon )$. The average of the logarithm of such counts for every vector for a particular dimension *m* is defined as $\phi (m)$. The ApEn for a particular dimension m and distance threshold, $\epsilon$, is $\phi (m)-\phi (m+1)$ [18]. The ApEn allows a template vector to be compared with itself (self-matching), which overestimates regularity. SampEn avoids self matching, which can make it more reliable for shorter time series [19, 20]. If ${\tilde{C}}_i^m(\epsilon )$ is the modified count of vectors without self matching, the SampEn is defined as $log\frac{{\tilde{C}}_i^m(\epsilon )}{{\tilde{C}}_i^{m+1}(\epsilon )}$. The permutation entropy(PE) constructs time delayed vectors, but considers the order in which the values appear rather than the actual magnitude. This is achieved by rank ordering the components of the vectors. The probability of occurrence of these rank-ordered vectors is used to calculate the PE of the time series [21, 22].

**Fig. 2:**
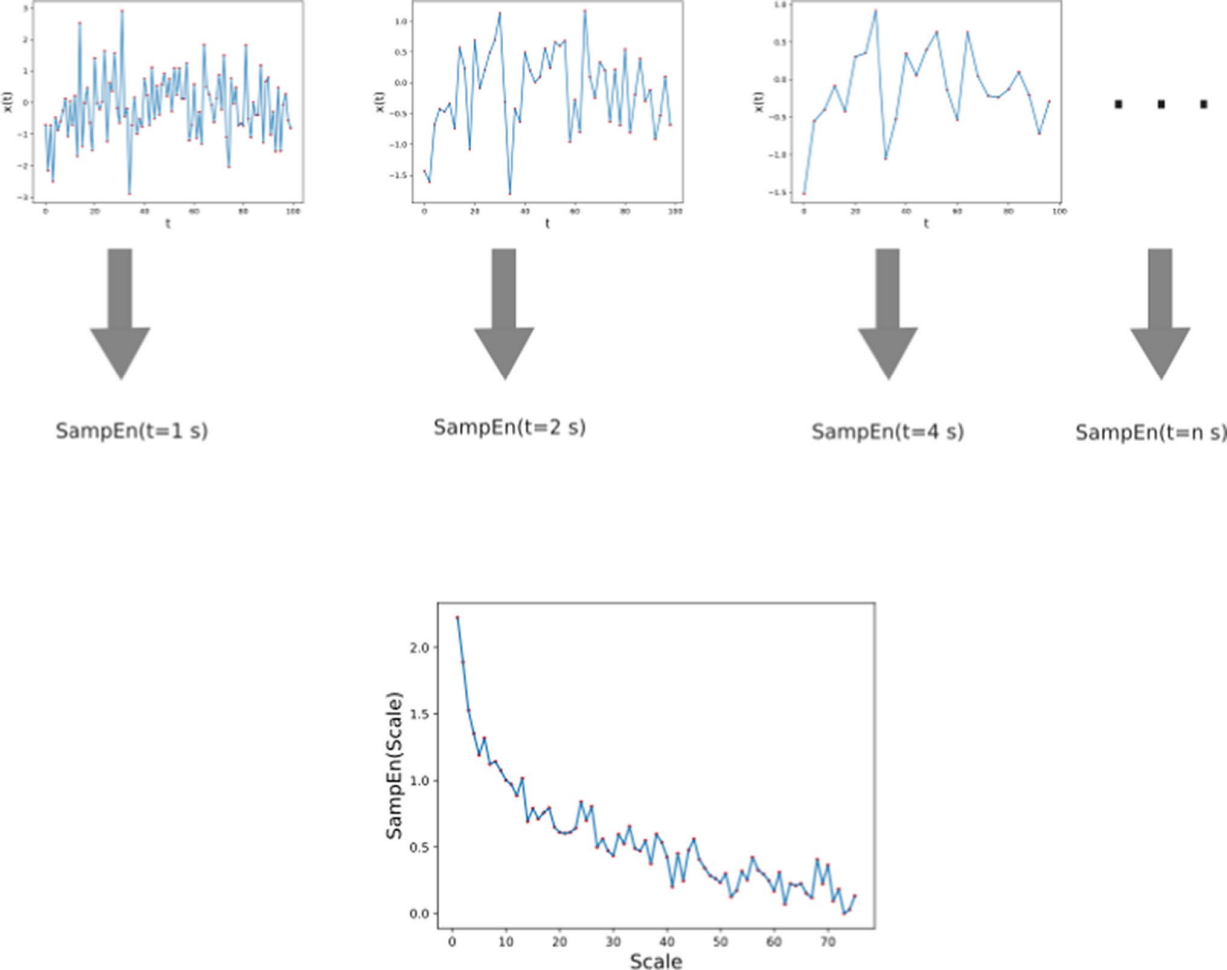
Schematic showing how the multiscale entropy (MSE) is calculated. A time series is resampled initially at different sampling rates, and the SampEn is calculated for each. The MSE is often calculated as the area under the scale vs SampEn curve

The multiscale entropy (MSE) generalizes SampEn to multiple time scales. To do this, first the time series is resampled by averaging at different sampling times. Doing this reduces fluctuations at lower time scales. The SampEn is calculated for each of these resampled time series. This determines the variation of fluctuations for distinct scales of measurement [23]. The MSE is often estimated as the area under the SampEn vs scale curve. A schematic for the MSE is shown in Figure 2. While SampEn is the most commonly used, multiscale methods can be used to generalize any entropy metric using the same procedure [24]. Finally, the Lempel–Ziv Complexity (LZC) converts a time series into a binary sequence and then counts the number of unique subsequences present in it [25, 26]. A brief overview of these information theory based metrics are given in Table 1.

**Table 1:** Calculation and definition of various information theory metrics. More details of calculation is shown in Supplementary 1. ApEn: Approximate Entropy, SampEn: Sample Entropy, MSE:Multiscale Entropy, PE:Permutation Entropy, LZC: Lempel-Ziv Complexity

Quantifier	Calculation	Definition
Shannon	Estimates the probability of values in the time series. Measures average uncertainty based on frequency of occurance	Measure of the uncertainty or randomness in the distribution of states.
ApEn	Constructs delay vectors, and counts likelihood of similar patterns of length *m*	Quantifies the regularity and unpredictability of time series data.
SampEn	Log of ratio of number of matching patterns of length m+1 to number of matching patterns of length m	Similar to ApEn, but eliminates self-matches.
MSE	Calculates entropy across different temporal scales by resampling the time series	Change in entropy over multiple time scales.
PE	Normalized Shannon entropy of ordinal patterns in subsequences of the time series	Complexity of temporal ordering in the time series.
LZC	Number of distinct patterns in a binary representation of the time series divided by its length	Compressibility and complexity of a time series.

### Metrics based on dynamical systems

In the above examples, where we used information theory based metrics, we made no assumptions about any underlying model. An alternate set of metrics for complexity are defined based on dynamical systems theory, where the underlying assumption is that the time series being analysed is derived from a set of differential equations $\dot{\vec {x}}=f(\vec {x})$. Such nonlinear dynamical systems can show a number of behaviors, including steady, periodic, and chaotic trajectories. The complexity of the dynamics of this system is measured using a number of metrics that quantify the space of its variables (state space) [27].

While this is obvious when all the variables are known, in any real time series there is an obvious gap, since only a single scalar time series is usually observed, whereas the state space of the underlying differential equations is n-dimensional. The fundamental assumption of nonlinear time series analysis is that the underlying n-dimensional state space of the set of differential equations can be reconstructed from the time series of one of its components. This makes intuitive sense, since the variables are coupled, i.e. changes in one of them affect the others. For instance, let us consider that the dynamics of three mood variables, say feeling sad, cheerful and suspicious, are governed by a set of differential equations. If we measure only one of these, it must have information about the variation of the others. The mathematics behind this is Taken’s embedding theorem. In essence it states that if we have a time series $X=\{x_1,x_2,...,x_n\}$, for a suitable delay time, $\tau$ and embedding dimension, *m*, we can reconstruct the vectors of its state space as $\vec {v_i}=\{x_i,x_{i+\tau }, ..., x_{i+m\tau }\}$. Taken’s theorem guarantees that the properties of this reconstructed space approximate those of the original system, as long as we use large enough *m* [28]. There are a number of methods to choose the delay time, $\tau$. In practice, all of them attempt to find the point at which the time series becomes uncorrelated with itself [29]. A simple measure is to find the time at which the autocorrelation falls to less than $\frac{1}{e}$. In Figure 1 (g)-(i) the reconstructed spaces in 2 dimensions for noisy, chaotic and periodic time series are presented. Patterns that are visible in the periodic and chaotic reconstructed spaces are absent in the noisy one.

**Fig. 3:**
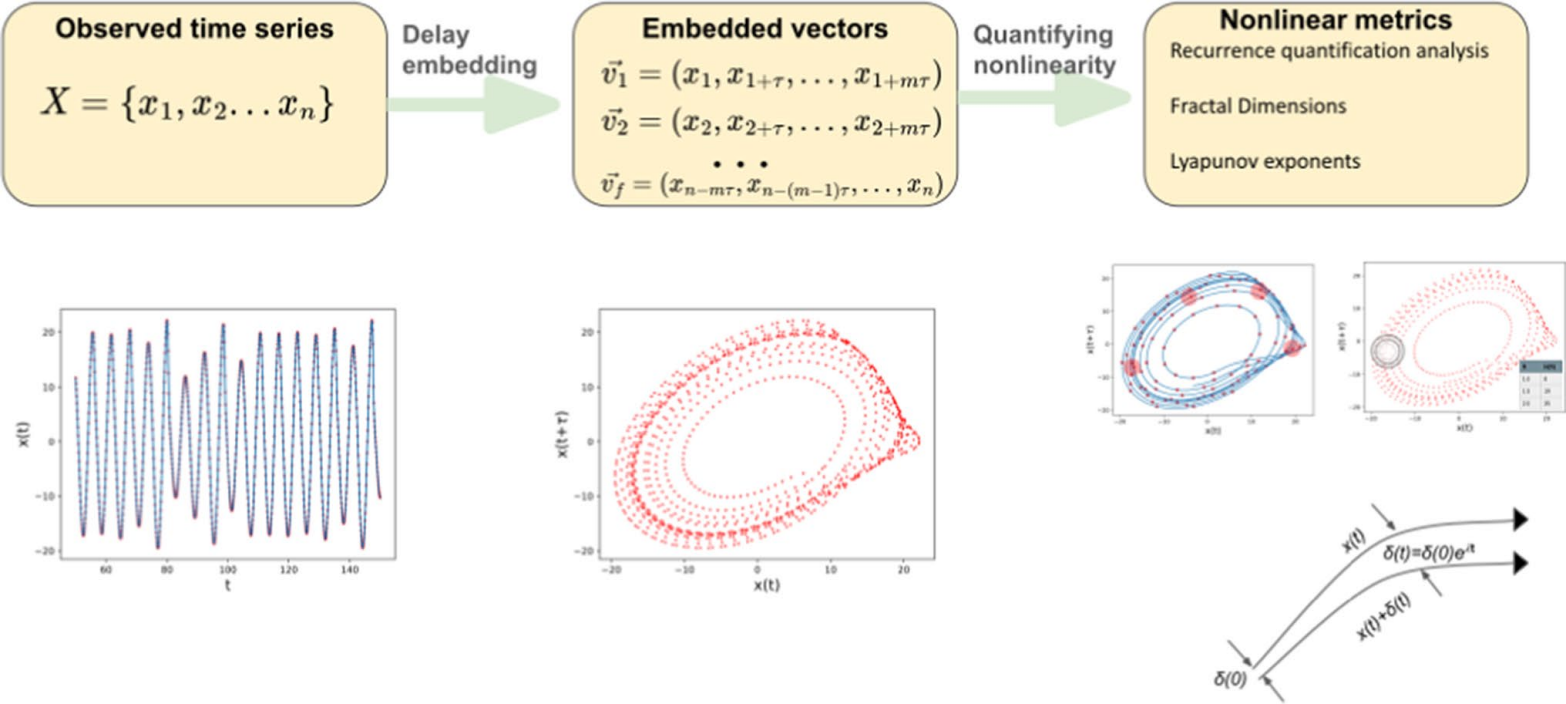
Overview of techniques using methods from nonlinear time series analysis

Once we have the reconstructed state space, a number of useful metrics can be quantified from it. These are based on properties of nonlinear dynamical systems. One relates to the recurrence of trajectories in this space, quantified using recurrence quantification analysis [30]. Another relates to the fractal structure of these trajectories in this space, quantified using fractal dimensions. A third relates to the exponential divergence of nearby trajectories in phase space, quantified using Lyapunov exponents. An overview of the methods in nonlinear time series analysis is shown in Figure 3.

*Recurrence quantification analysis (RQA)*: Since the trajectories of dynamical systems are bounded, even when the trajectories are not periodic, they must recur arbitrarily close to each other. RQA quantifies the recurrence of trajectory points within a neighborhood of a point in phase space. This process is conducted for all points, and an $n \times n$ matrix, which is visualized as a recurrence plot. When the dynamics is purely periodic, the recurrence plot shows long diagonal lines. When the system is stuck at an equilibrium, the plot shows long horizontal/vertical lines. If the trajectories are purely noisy, points are randomly scattered in the plot. Hence quantifying the distribution of lines in the recurrence plot helps us discern the periodicities, points where the dynamical system spends more time and the stochasticity in the system [30]. A key advantage of RQA is the ability to capture recurrent behavior that is not strictly periodic. For example, in the context of physical activity, RQA can effectively quantify patterns in an individual’s biking habits, even if they ride only a few times a month without a regular schedule. Other methods like power spectral analysis may fail to detect such non-periodic recurrent patterns. Common metrics derived from the recurrence plot are briefly described in Table 2.

**Table 2:** Calculation and definition of various recurrence plot quantifiers [31]. *DET*: Determinism; *LAM*: Laminarity; $L_{avg}$: Average diagonal line length; $L_{ent}$: Entropy of diagonal lines; *TT*: Trapping time; $V_{ent}$: Entropy of vertical lines

Quantifier	Calculation	Definition
*DET*	Fraction of recurrent points forming diagonal lines	Level of deterministic activity in the data.
*LAM*	Fraction of recurrent points forming vertical lines	Level of slowly evolving processes in the time series.
$L_{avg}$	Average length of diagonal lines	Average duration of repeating patterns
$L_{ent}$	Entropy of diagonal line distribution	Range of durations of repeating patterns
*TT*	Average length of vertical lines	Average duration of static patterns
$V_{ent}$	Entropy of vertical line distribution	Range of durations of static patterns.

*Fractal dimensions*: Fractal dimensions are a measure of the dimension of the space in which the dynamics of the system occurs. This is different from the dimensionality of the system, which reflects the number of variables in the system. A dynamical system with three variables could exhibit purely periodic dynamics (limit cycle), represented by a 1-d closed loop in phase space (Figure 1 c and f). For certain value of parameters, the system could show chaotic dynamics, like what we see in Figure 1h. Unlike periodic dynamics which appear in a neat loop, or noisy dynamics which appear completely random, chaotic dynamics result in intricate patterns in phase space that are self-similar, and are called ’strange attractors’. This self-similarity is captured using a fractal dimension. In nonlinear time series analysis, a non-integer fractal dimension is taken as being indicative of chaos in the system [32]. However, the presence of noise can also result in the calculated fractal dimensions having non-integer values in processes that are not chaotic [33].

Different parts of the strange attractor may have different values locally. The multifractal spectrum generalizes the idea of a fractal dimension to a spectrum of dimensions which describe the fractality of the attractor locally.

A number of measures of the fractal dimension exist, such as the Hausdroff dimension, box counting dimension, information dimension and correlation dimension [34]. In addition, quantifiers such as the Higuchi dimension and Kantz dimension measure the fractality in a time series without embedding it [35, 36]. A closely related measure is the Hurst exponent, which measures the long term fluctuations in the time series, and is normally estimated through detrended fluctuation analysis (DFA) [37]. The Hurst exponent takes a value of 0.5 for white noise, with values greater than 0.5 indicating longer term memory. The Hurst exponent is related to the fractal dimension through the formula $D=2-H$ [38]. Many of these are generalized to generate corresponding multifractal spectra [27, 39, 40].

*Lyapunov exponents* : One way to detect chaos in a system is to see how trajectories that are very close to each other evolve in time. When a system is chaotic, these trajectories would exponential diverge away from each other.The Lyapunov exponent measures this divergence of nearby trajectories in phase space, and is the true measure of chaos in a dynamical system. If two trajectories have an initial separation $\delta (0)$ and a separation of $\delta (t)$ after a time t, the Lyapunov exponent, $\lambda$ measures the exponential divergence of the trajectories in a particular direction, $\delta (t)\approx e^{\lambda t}\delta (0)$. Hence there is a spectrum of Lyapunov exponents corresponding to the direction being considered. For a periodic time series the largest of these Lyapunov exponents (LLE) will be 0, while for chaotic dynamics, there will be at least one positive exponent [27, 34].

A number of algorithms, such as the Rosenstein algorithm, Kantz algorithm, and modifications of these, estimate the Lyapunov exponent from time series [24, 41, 42]. However, in real time series affected by noise, finding the true value of the Lyapunov exponent can be complicated. Addition of noise to chaotic time series can result in reduced values for the maximal exponent, whereas noise can also show spurious positive exponents for non chaotic time series [43, 44]. Hence, caution must be taken while interpreting values of Lyapunov exponents from real time series.

## Variations in complexity metrics

In this section we show how the metrics of time series complexity described above vary as the dynamics become more noise dominated, and as it becomes more periodic. To understand these changes, we simulate data from a Rössler system, a well studied nonlinear dynamical system that exhibits a range of dynamical behavior including chaos [45]. The Rössler is a good system to study the effects of changes in complexity metrics, since it has a strong periodic component and exhibits a period doubling route to chaos, progressively moving from purely periodic dynamics into chaotic dynamics. This is useful in the context of real data where increased rhythmicity or stochasticity in the time series could be indicative of pathology.

In our investigation, we start the dynamics of the Rössler system from a period-1 case ($a=0.1,b=0.1,c=4.0$). We then progressively change the complexity to a period-8 case ($a=0.1,b=0.1,c=8.7$), and to chaos ($a=0.1,b=0.1,c=14.0$).We then consider the case where the system becomes more noisy. While systems can become more noisy through a number of different ways, we consider only the simplest, i.e. additive Gaussian white noise. We add noise to the time series of the Rössler in the chaotic regime, progressively increasing the level of added noise from $1\%$ to $5\%$ to $10\%$. The percentages represent the relative standard deviation of the noise and the original Rössler time series. The metrics are then calculated for each and the results are presented in Table 3.

Most entropy metrics and fractal dimensions increase as the dynamics become more stochastic, while most recurrence based metrics decrease with stochasticity. A few metrics peak for chaotic dynamics, and taper off if the dynamics is more periodic or stochastic (shown in bold). Other metrics, such as the LLE suffer from high sensitivity to noise, and show abnormally high values when data becomes overly noise contaminated. Hence, different metrics capture different aspects of complexity, with some increasing as the data becomes more noisy, and others peaking when the system is chaotic. In this study, we see that the Shannon entropy, TT and $D_{ent}$ peaks for chaos. Of these, the Shannon entropy is not dependent on temporal ordering, and will increase for any process whose amplitude distribution deviates from a flat distribution. A combination of metrics can be used to determine the likely effect that a disorder has on the dynamics of the time series.

All the simulations were conducted in Python v3.11.3, using the scipy and numpy libraries [46, 47]. The metrics were calculated using the neurokit2 and HFDA packages [24]. The code for this simulation is provided in https://github.com/sgeorge91/complexity-metrics-of-time-series.

**Table 3:** Change in complexity metrics as the dynamics of the Rössler changes from periodic to chaotic to noisy dynamics. The quantifiers which show maximum value for the chaotic time series are marked in bold. ApEn: Approximate Entropy, SampEn: Sample Entropy, MSE: Multiscale Entropy, PermEn: Permutation Entropy, LZC: Lempel-Ziv Complexity, DET: Determinism, LAM: Laminarity, $D_{avg}$: Average diagonal line length, *TT*: Trapping time, $D_{ent}$: Entropy of diagonal line lengths, $V_{ent}$: Entropy of vertical line lengths, HD: Higichi Dimension, KD: Katz Dimension, $D_2$: Correlation dimension, *LLE*: Largest Lyapunov Exponent

Metric	Period-1	Period-8	Chaos	1%	5%	10%	Noise	Direction
**Shannon**	**2.22**	**2.29**	**2.31**	**2.31**	**2.30**	**2.27**	**1.59**	**Inconsistent**
ApEn	0.27	0.34	0.38	0.39	0.46	0.66	2.20	Increase
SampEn	0.24	0.32	0.36	0.37	0.44	0.61	2.20	Increase
MSE	0.065	0.082	0.091	0.091	0.091	0.092	0.07	Inconsistent
PermEn	0.01	0.01	0.01	0.02	0.07	0.18	0.30	Increase
LZC	0.01	0.04	0.06	0.06	0.10	0.14	1.03	Increase
DET	1.00	1.00	0.99	0.99	0.94	0.89	0.71	Decrease
LAM	1.00	1.00	1.00	0.99	0.98	0.94	0.31	Decrease
$D_{avg}$	56.25	33.66	27.10	25.03	13.78	8.06	2.86	Decrease
**TT**	**6.00**	**6.06**	**6.83**	**6.83**	**6.71**	**6.23**	**2.45**	**Inconsistent**
$\mathbf {D_{ent}}$	**2.53**	**3.30**	**3.33**	**3.26**	**2.66**	**2.28**	**1.28**	**Inconsistent**
$V_{ent}$	0.86	1.75	2.16	2.16	2.21	2.25	0.90	Inconsistent
HD	1.02	1.02	1.02	1.02	1.06	1.18	2.00	Increase
KD	2.77	2.97	3.02	3.02	3.10	3.41	5.87	Increase
$D_2$	1.01	1.58	1.80	1.80	1.95	2.20	2.46	Increase
Hurst	0.218	0.241	0.258	0.258	0.259	0.262	0.492	Increase
LLE	0.000	0.005	0.006	0.006	0.004	0.003	0.427	Inconsistent

The quantifiers which show maximum value for the chaotic time series are marked in bold

## Studies on depression

The study of complexity metrics provides valuable insights into the nonlinear dynamics underlying depression. This section reviews findings from various domains, including EEG, EMA, ECG, and other behavioral data, highlighting the diverse ways complexity is altered in the context of depression. We mainly examine studies in three time series measurements, namely actigraphy, electroencephalogram (EEG) and electrocardiogram (ECG). These were chosen since they satisfy some of the underlying assumptions of the time series complexity measures mentioned in this paper, namely evenly sampled long time series with high sampling frequency. Consequently, studies using the complexity metrics mentioned above are more common in these.

### Actigraphy

We first investigate results reported on the complexity of physical activity measurements in relation to depression. In the context of actigraphy, complexity metrics quantify the properties of the patterns present in the data, including the influence of sleep and circadian rhythms. Actigraphy data analyzed using recurrence quantification analysis (RQA) revealed that depressed individuals exhibit reduced duration and diversity of recurrent activity patterns, with an increased $D_{avg}$ and $D_{ent}$ in the RP, and a decreased $\frac{LAM}{DET}$ [31]. Similarly, the Hurst exponent of spontaneous motor hand activity showed higher values in depressed individuals as compared to controls. This increase was interpreted as an indication of “trivial complexity,” potentially reflecting random fluctuations or movements [48].

Relatively few studies exist on the complexity of actigraphy data for depression. Taken together, the findings above suggest that depression is associated with a loss of structured activity patterns which could be potentially due to a combination of sleep disturbances, psychomotor slowing and diminished motivation. This interpretation complements other studies that indicate high variability of activity patterns and disturbed circadian rhythms, suggesting that depression leads to a decrease in complexity due to increased noise [49–52]. In line with our results in Section 3, this would lead to decreases in RP metrics and increases in many entropy metrics.

### EEG studies

The complexity analysis of EEG data has been extensively applied to understand depression dynamics [53]. Fractal analysis of EEG signals has revealed higher Higuchi fractal dimension values in single-channel EEG, as well as those recorded from the frontal and parietal lobes of depressed patients compared to non-depressed individuals [54, 55]. Additionally, depressed individuals often exhibit higher values for Shannon entropy, LZC, and Kolmogorov complexity [56–58]. These studies are consistent with either EEG from depressed individuals showing more stochastic or chaotic dynamics as compared to non-depressed groups. This would indicate a greater unpredictability in neural activity due to depression, resulting in diminished mood regulations.

Nevertheless, findings are not always consistent [14]. Other research has revealed a more nuanced pattern, where some metrics, such as SampEn, multiscale LZC, LLE, fractal dimensions, and bispectral features, showed higher values in the control group, while others, including DET, LAM, recurrence times, $D_{ent}$, and the Hurst exponent, were significantly elevated in depressed individuals [59, 60]. In our simulations, these trends are not consistent with increasing noise or with chaos. This could indicate a more complex pattern, with higher variability between different patient groups and symtom severity. Moreover, EEG signals are typically more complicated signals composed of multiple frequency bands corresponding to different brain regions and mental states. This could result in greater sensitivity to preprocessing methods and parameter choices.

### ECG studies

ECG data is another context in which complexity metrics have been used extensively to study variations as a result of depression. Both heart rate variability (HRV) and its complexity are known to be lower in depressed individuals as compared to healthy individuals [61]. This reduction is interpreted as being indicative of higher cardiac sympathetic activity and more rigid parasympathetic modulation [62, 63]. The entropy in particular has been reported as being an good measure to detect depression, with many measures including Shannon entropy and ApEn showing lower values in depressed groups compared to controls [64–66]. Similarly, a diminished LLE has also been reported in individuals with depression [67]. The power spectrum slope, which is closely related to the Hurst exponent, was also seen to be reduced in the depressed group compared to the non-depressed [68]. Overall, these differences are consistent with depression reducing the chaotic dynamics in ECG, possibly due to increased periodicity leading to reduced emotional reactivity of the heart. However, as in the case of EEG data, the picture may be more complex, with some studies indicating increased fractal dimensions and entropy measures for subclinical depression and in individuals at risk for depression recurrence [69, 70]. This indicates that depression stage and severity could affect the complexity of cardiac response.

### Other studies

Beyond these, fewer studies have examined the complexity of depression in other types of time series, such as ecological momentary assessment (EMA). In the context of EMA, studies have consistently found complexity metrics indicative of nonlinearity [6, 71, 72], although to the best of our knowledge no studies have systematically examined how this complexity changes as a result of depression. However, since the temporal dynamics of mood and behavior are governed by complex processes, it is possible that they may shift in the presence of depression.

Beyond EEG, magnetoencephalography (MEG) measurements have also indicated increased LZC in depressed individuals compared to healthy controls. This value reduced after effective pharmacological treatment [73]. Studies of complexity in functional magnetic resonance imaging (fMRI) studies have suggested a higher MSE in depressed individuals as compared to healthy controls in data from the left fronto-parietal network. However, the results were shown to be heavily dependent on the chosen parameters for calculations [74]. A summary of the findings from literature for the three main time series considered in this section are described in Table 4.

**Table 4:** Summary of findings in changes in complexity in depression across actigraphy, EEG and ECG measures

Measurement	Metrics reported	Direction of change	Interpretation	References
Actigraphy	RQA (DET, LAM, $D_{avg}$, $D_{ent}$), Hurst exponent	$\downarrow$ DET, LAM; $\uparrow$ $D_{avg}$, $D_{ent}$, Hurst	Increased noise and irregularity possibly due to sleep disturbances or low motivation	[31, 48]
EEG	Shannon Entropy, LZC, Kolmogorov complexity, Fractal dimensions, LLE, RQA	$\uparrow$ Shannon, LZC, Higuchi FD; mixed trends in SampEn, LLE, RQA, MLZC	Inconsistent results; may depend on stage and severity	[54–60]
ECG (HRV)	Shannon Entropy, ApEn, LLE, Power spectrum slope, Hurst exponent	$\downarrow$ Entropy, LLE, Hurst; occasional $\uparrow$ in FD	Lower complexity due to increased periodicity; suggests reduced adaptive capacity	[61, 64–68]

## Discussion

In this section the main takeaways from this perspective article are summarized. The limitations of studies on complexity in depression, specially in the context of the approach taken in this article, are then outlined. Finally, several promising directions for future research on complexity in depression are proposed.

### Summary

In this perspective article we examine what is meant by time series complexity and what aspects of complexity is measured using various metrics of time series complexity. We start with an overview of complexity metrics evaluated from time series, included common metrics from information theory and nonlinear time series analysis. We then examine how these change when the dynamics of the time series is periodic, chaotic or noisy. We identify that most entropy metrics and fractal dimensions show highest values for purely noisy time series, while RQA based metrics show highest values for periodic time series. Three metrics were identified which show maximum value for chaotic time series, namely Shannon entropy, TT and $D_{ent}$. Of these, however, the Shannon entropy depends solely on the amplitude distribution of the time series, and hence will take different values for a different dynamical system or a different type of noise.

These different time series metrics are designed to capture different aspects of complexity. Information theory based metrics are designed to study information content in time series, and as such work well to distinguish periodic patterns from noise [75]. These do not take dynamics explicitly into consideration, unlike measures based on dynamical systems. Measures based on dynamical systems have been explicitly designed to distinguish between the dynamical behaviors shown by nonlinear dynamical systems [34]. However, these are designed for purely deterministic systems, and are more complicated to interpret in the presence of noise. RQA, though based on nonlinear dynamics, offers a range of metrics that quantify periodic and equilibrium patterns in time series [30]. Hence, these different metrics capture different aspects of time series complexity.

We then studied the values reported for these metrics for actigraphy, EEG and ECG time series derived from depressed groups and how they compare to similar metrics reported for control groups. Studies on actigraphy suggested that the dynamics becomes more stochastic as a result of depression. This suggests that depression may be associated with less structured and more fragmented movement patterns. On the other hand results on EEG data showed inconsistent trends, with some studies indicating that depression results in more stochastic or chaotic dynamics, while others do not indicate any clear trend. We believe that this could be inherent in the nature of EEG time series, which are composed of multiple frequency bands corresponding to different functions. Studies on the ECG suggested depression leads to a reduction in complexity due to increased periodicity. This reduction could indicative of diminished responsiveness to emotional demands. However, studies on related groups, such as subclinical depression or individuals at risk for depression showed different trends, including increased fractal dimensions and entropy. This could indicate that changes in the cardiac complexity is dependent on the stage and severity of depression.

One of the main reasons for studying complexity changes in psychopathology is to discover objective markers of disease. In this study, we explored a range of metrics from information theory and nonlinear dynamical systems. Ultimately, the choice of metric depends on practical considerations about the time series, and the direction of changes that are expected from clinical knowledge. Metrics that involve delay embedding or analysis over different scales (multiscale methods) could be ill suited to time series that are short. Shortness depends on the time scale of the data, and the underlying process. For instance, to capture effects of depression on actigraphy, the time series under consideration should span multiple days to fully capture the variability between days. Alternatively, we may expect specific characteristics of the disease to manifest in a particular measurement. For example, increased affective inertia is thought to characteristic of depression [76, 77]. This is likely to result in increased LAM (which measures such slowing), in EMA data. Hence the choice of what metric should be used for a specific time series should be informed by the effects that are hypothesized to occur and limitations of the time series.

Studies on time series complexity are difficult to navigate, since there are multiple interpretations to what complexity implies. Different metrics quantify different aspects of this complexity, resulting in conflicting interpretations. Moreover, different metrics are formulated in the context of changes in dynamics relevant to a specific problem. For instance, metrics from nonlinear time series analysis are designed to study changes in dynamical states and can distinguish between chaos and periodicity. However, they are ill posed to study stochastic dynamics or even dynamics with modest noise contamination [78]. This article attempts to start addressing this problem by studying how commonly used complexity metrics change as the dynamics becomes more stochastic or periodic.

### Limitations

While the question itself is important, the treatment taken in this article is quite rudimentary. For one it studies only a particular type of noise, namely additive white noise. A more realistic scenario would consider stochastic differential equations, systems that evolve with noise. Another approach that is often more relevant for time series that are measured infrequently, such as EMA, is using difference equations or maps, where each time point is considered as some function of previous time points ($x_i=f(x_{i-1},x_{i-2},...x_{i-n})$). In such cases, other metrics, such as the transition probabilities between states, or stability of states may be quite relevant to study [79]. In fact, linear maps studied using autoregressive models are a common approach to study EMA time series in psychology [80, 81]. Moreover, in this study we used only a specific system, namely the Rössler, and added noise only to the chaotic case. Noise contaminated periodicity can be confused for chaos, and a more thorough investigation would include these dynamical states as well [15]. However, such approaches are likely to lead to more complex changes in the metrics being studied, and a range of scenarios that could lead to the same conclusions.

A limitation of this study, and of studies in time series complexity in general, is that the choice of parameters for evaluating these metrics greatly influence results. Much research has been dedicated into finding the ideal set of parameters for each of these metrics, but real world time series can be plagued by issues such as noise, uneven sampling or limited data length, which could lead to surprising results [29, 82]. These issues are aggravated in psychological time series such as EMA, where requirements of even sampling times and long sampling durations, are often not met [83]. The relatively few studies on the complexity of EMA ratings in the context of depression could be due to these limitations. In these cases, other measures such as dynamic complexity have been developed which are relatively robust to these issues [84, 85]. On the other hand, when the metrics mentioned in this article are utilized for under-sampled or short time series, it is important to investigate their tolerance before making conclusions about the underlying mechanism for observed changes.

A final limitation in the approach in this perspective is the treatment of depression in binaries, i.e either as depressed or non-depressed. In reality, as mentioned in the introduction depression varies greatly in the range and severity of symptoms. These can contribute in different ways to complexity, and has been mentioned by other authors as reasons for the inconsistent results observed in EEG time series [14]. However, studies on complexity in depression are limited, and studies that examine differences within depression are fewer. Studies that have examined this relationship in EEG have found a strong positive correlation between symptom severity and complexity (LZC) [86]. Moreover, the fractal dimension and entropy of ECG were found to be higher in subclinical depression, in contrast to studies in groups with clinical depression, again suggesting that symptom severity could affect observed complexity patterns [69].

### Future directions

Depression is a complex disease that is known to affect a range of different responses in the body. Apart from those mentioned above, it affects other measurements such as hormone levels, immune responses and gait [87–89]. Complexity metrics have been used to study many of these as well, though studies specific to depression are few [90–92]. One focus of future studies could be to quantify how depression affects the complexity in these measurements. Even among measurements that are relatively common, such as EEG, a more detailed understanding of the effect of depression on complexity would require further investigation. These would involve more specific mathematical models and other types of noise.

Changes in complexity across different measurements, if correctly identified, could serve as objective markers of instability, which could be used as potential digital biomarkers of depression [93, 94]. With the growth of wearable devices that measure actigraphy and ECG, markers in these measurements can aid in ambulatory monitoring of symptoms [95]. In the case of ECG, for instance recent studies have shown that changes in variability and complexity metrics could precede the onset of depression [63, 70]. If verified in a large sample, this offers a potential way forward to predict this onset of depression. It is also likely that combinations of complexity metrics across measurements could offer a more comprehensive prediction of depression onset.

In summary, this article provides a simple framework for understanding the behavior of complexity metrics across dynamical regimes, and is intended as a starting point for future researchers looking to interpret what their results could imply for the mechanism underlying the pathology that they are investigating. However, it also underscores the need for a more nuanced, context-specific investigation. A deeper exploration into the interplay between noise, dynamics, and complexity metrics will be essential to advance our understanding of time series complexity, particularly in the context of psychopathology.

## Supplementary Information

Below is the link to the electronic supplementary material.

Supplementary file 1.

## Data Availability

No datasets were generated or analysed during the current study.
