# Supplementary material for: Chaos to clarity: interpreting time series complexity metrics with an application to depression
Source: Discov Ment Health. 2025 Jul 1;5(1):97. doi: 10.1007/s44192-025-00231-4 (PMC12214179; doi:10.1007/s44192-025-00231-4)
Supplement: Supplementary file 1 [file 44192_2025_231_MOESM1_ESM.pdf]

Supplementary material to “Chaos to Clarity: Interpreting time series complexity metrics with an application to depression”

Sandip V George[1]

[1]The Institute for Complex Systems and Mathematical Biology, Department of Physics, University of Aberdeen, Aberdeen, UK

Table 1: Calculation of various information theory metrics.

Quantifier	Calculation
**Shannon Entropy**	• Time series: $X = \{x_1, x_2 ... x_n\}$<br>• Shannon Entropy: $H(X) = -\sum_{i=1}^{n} p(x_i) \log p(x_i)$
**Approximate Entropy (ApEn)**	• Template vectors: $\vec{v_i} = (x_i, x_{i+1}, ..., x_{i+m-1})$<br>• Count of vectors in an ϵ neighborhood: $C_i^m(\epsilon)$<br>• $$\phi^m(\epsilon) = \frac{1}{N-m+1} \sum_{i=1}^{N-m+1} \log C_i^m(\epsilon)$$<br>• $$\text{ApEn}(m, \epsilon) = \phi^m(\epsilon) - \phi^{m+1}(\epsilon)$$
**Sample Entropy (SampEn)**	• Count of vectors in an ϵ neighborhood without self matching: $\tilde{C_i}^m(\epsilon)$<br>• $$\text{SampEn}(m, \epsilon) = -\log\left(\frac{\tilde{C}_i^m(\epsilon)}{\tilde{C}_i^{m+1}(\epsilon)}\right)$$
**Multiscale Entropy (MSE)**	$$\text{MSE} = \text{SampEn computed on coarse-grained time series}$$<br>where coarse-graining is performed at multiple scales by averaging non-overlapping segments of the time series.
**Permutation Entropy (PE)**	$$H_{\text{PE}} = -\sum_{\pi} p(\pi) \log p(\pi)$$<br>where $p(\pi)$ is the probability of an ordinal pattern π occurring in a time series segment.
**Lempel-Ziv Complexity (LZC)**	$$\text{LZC} = \frac{c(n)}{n}$$<br>where $c(n)$ is the number of unique substrings in a binary sequence of length n.

ApEn: Approximate Entropy, SampEn: Sample Entropy, MSE: Multiscale Entropy, PE: Permutation Entropy, LZC: Lempel-Ziv Complexity

Table 2: Calculation of metrics from dynamical systems

Quantifier	Calculation
**Box-Counting**	• Embed time series: $\vec{v_i} = (x_i, x_{i+\tau}, ..., x_{i+(m-1)\tau})$<br>• Cover embedded space with hypercubes of size ϵ<br>• Count number of occupied boxes: $N(\epsilon)$<br>• Estimate dimension: $D = -\lim_{\epsilon \to 0} \frac{\log N(\epsilon)}{\log \epsilon}$
**Correlation Dimension** (D_2)	• Embed time series as above<br>• Correlation sum: $C(\epsilon) = \frac{2}{N(N-1)} \sum_{i<j} \Theta(\epsilon - \lVert \vec{v_i} - \vec{v_j} \rVert)$<br>• Estimate dimension: $D_2 = \lim_{\epsilon \to 0} \frac{\log C(\epsilon)}{\log \epsilon}$
**Higuchi Dimension(HD)**	• Construct k subseries: $X_k^m = \{x_m, x_{m+k}, x_{m+2k}, \dots\}$<br>• Compute length $L(k)$ of curve for each k<br>• Fit $L(k) \propto k^{-D}$ to get dimension D
**Hurst Exponent (via DFA)**	• Integrate signal: $Y(k) = \sum_{i=1}^{k} (x_i - \bar{x})$<br>• Divide into segments, detrend each segment linearly<br>• Compute RMS fluctuation $F(s)$ for segment size s<br>• Estimate: $F(s) \propto s^H$, then $D = 2 - H$
**Lyapunov Exponent (Rosenstein)**	• Embed: $\vec{v_i} = (x_i, x_{i+\tau}, ..., x_{i+(m-1)\tau})$<br>• For each $\vec{v_i}$, find nearest neighbor $\vec{v_j}$ with $\lvert j - i \rvert > W$<br>• Track divergence: $d_i(t) = \lVert \vec{v_{i+t}} - \vec{v_{j+t}} \rVert$<br>• Estimate LLE from slope of $\langle \log d_i(t) \rangle$ vs t
